# Bromide of Strontium in the Treatment of Epilepsy

**Published:** 1898-10-29

**Authors:** 


### BROMIDE OF STRONTIUM IN THE TREATMENT OF EPILEPSY.

Dr. A. Roche* again urges the use of bromide of strontium in epilepsy. He says that the strontium salt has an immense advantage over that of potassium, which has frequently produced serious physical and mental changes when continued for long periods. Moreover, patients taking bromide of potassium frequently suffer from intense depression, sometimes, in fact, asserting that they had rather suffer from the fits than from the depression produced by the medicine. This consequence has not been noticed at all in the cases taking bromide of strontium. Dr. Roche has given three drachms daily for weeks without any unpleasant symptoms, and he considers that it excels the bromide of potassium in being more efficient; in not being followed by dangerous consequences when it is continued over a lengthened period; and in that, as it is not poisonous, it can be given in as large a dose as may be required to control the attacks. He usually commences the treatment by ordering half a drachm night and morning in some vegetable tonic infusion, and if that does not control the attacks he rapidly increases the dose till he has found the quantity which will suit the individual case. Where there is any warning of the attack he directs the patient to take 30 grains at once, and to repeat it every hour if required. Diet, however, is of much importance, and it is well that the patients should be restricted to fish and vegetables; although, when the attacks diminish in number, the more easily digested meats may be allowed twice or three times in the week.

